# Machine Learning in Agriculture: A Review

**DOI:** 10.3390/s18082674

**Published:** 2018-08-14

**Authors:** Konstantinos G. Liakos, Patrizia Busato, Dimitrios Moshou, Simon Pearson, Dionysis Bochtis

**Affiliations:** 1 Institute for Bio-Economy and Agri-Technology (IBO), Centre of Research and Technology—Hellas (CERTH), 6th km Charilaou-Thermi Rd, GR 57001 Thessaloniki, Greece; k.liakos@certh.gr (K.G.L.); dmoshou@auth.gr (D.M.); 2 Department of Agriculture, Forestry and Food Sciences (DISAFA), Faculty of Agriculture, University of Turin, Largo Braccini 2, 10095 Grugliasco, Italy; patrizia.busato@unito.it; 3 Agricultural Engineering Laboratory, Faculty of Agriculture, Aristotle University of Thessaloniki, 54124 Thessaloniki, Greece; 4 Lincoln Institute for Agri-food Technology (LIAT), University of Lincoln, Brayford Way, Brayford Pool, Lincoln LN6 7TS, UK, spearson@lincoln.ac.uk

**Keywords:** crop management, water management, soil management, livestock management, artificial intelligence, planning, precision agriculture

## Abstract

Machine learning has emerged with big data technologies and high-performance computing to create new opportunities for data intensive science in the multi-disciplinary agri-technologies domain. In this paper, we present a comprehensive review of research dedicated to applications of machine learning in agricultural production systems. The works analyzed were categorized in (a) crop management, including applications on yield prediction, disease detection, weed detection crop quality, and species recognition; (b) livestock management, including applications on animal welfare and livestock production; (c) water management; and (d) soil management. The filtering and classification of the presented articles demonstrate how agriculture will benefit from machine learning technologies. By applying machine learning to sensor data, farm management systems are evolving into real time artificial intelligence enabled programs that provide rich recommendations and insights for farmer decision support and action.

## 1. Introduction

Agriculture plays a critical role in the global economy. Pressure on the agricultural system will increase with the continuing expansion of the human population. Agri-technology and precision farming, now also termed digital agriculture, have arisen as new scientific fields that use data intense approaches to drive agricultural productivity while minimizing its environmental impact. The data generated in modern agricultural operations is provided by a variety of different sensors that enable a better understanding of the operational environment (an interaction of dynamic crop, soil, and weather conditions) and the operation itself (machinery data), leading to more accurate and faster decision making.

Machine learning (ML) has emerged together with big data technologies and high-performance computing to create new opportunities to unravel, quantify, and understand data intensive processes in agricultural operational environments. Among other definitions, ML is defined as the scientific field that gives machines the ability to learn without being strictly programmed [1]. Year by year, ML applies in more and more scientific fields including, for example, bioinformatics [2,3], biochemistry [4,5], medicine [6,7,8], meteorology [9,10,11], economic sciences [12,13,14], robotics [15,16], aquaculture [17,18], and food security [19,20], and climatology [21].

In this paper, we present a comprehensive review of the application of ML in agriculture. A number of relevant papers are presented that emphasise key and unique features of popular ML models. The structure of the present work is as follows: the ML terminology, definition, learning tasks, and analysis are initially given in Section 2, along with the most popular learning models and algorithms. Section 3 presents the implemented methodology for the collection and categorization of the presented works. Finally, in Section 4, the advantages derived from the implementation of ML in agri-technology are listed, as well as the future expectations in the domain.

Because of the large number of abbreviations used in the relative scientific works, Table 1, Table 2, Table 3 and Table 4 list the abbreviations that appear in this work, categorized to ML models, algorithms, statistical measures, and general abbreviations, respectively.

## 2. An Overview on Machine Learning

### 2.1. Machine Learning Terminology and Definitions

**Figure 1:**
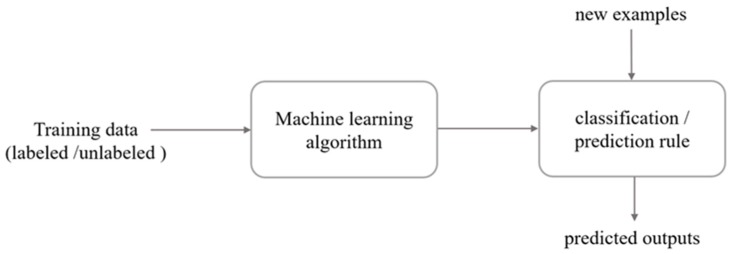
A typical machine learning approach.

Typically, ML methodologies involves a learning process with the objective to learn from “experience” (training data) to perform a task. Data in ML consists of a set of examples. Usually, an individual example is described by a set of attributes, also known as features or variables. A feature can be nominal (enumeration), binary (i.e., 0 or 1), ordinal (e.g., A+ or B−), or numeric (integer, real number, etc.). The performance of the ML model in a specific task is measured by a performance metric that is improved with experience over time. To calculate the performance of ML models and algorithms, various statistical and mathematical models are used. After the end of the learning process, the trained model can be used to classify, predict, or cluster new examples (testing data) using the experience obtained during the training process. Figure 1 shows a typical ML approach.

ML tasks are typically classified into different broad categories depending on the learning type (supervised/unsupervised), learning models (classification, regression, clustering, and dimensionality reduction), or the learning models employed to implement the selected task.

### 2.2. Tasks of Learning

ML tasks are classified into two main categories, that is, supervised and unsupervised learning, depending on the learning signal of the learning system. In supervised learning, data are presented with example inputs and the corresponding outputs, and the objective is to construct a general rule that maps inputs to outputs. In some cases, inputs can be only partially available with some of the target outputs missing or given only as feedback to the actions in a dynamic environment (reinforcement learning). In the supervised setting, the acquired expertise (trained model) is used to predict the missing outputs (labels) for the test data. In unsupervised learning, however, there is no distinction between training and test sets with data being unlabeled. The learner processes input data with the goal of discovering hidden patterns.

### 2.3. Analysis of Learning

Dimensionality reduction (DR) is an analysis that is executed in both families of supervised and unsupervised learning types, with the aim of providing a more compact, lower-dimensional representation of a dataset to preserve as much information as possible from the original data. It is usually performed prior to applying a classification or regression model in order to avoid the effects of dimensionality. Some of the most common DR algorithms are the following: (i) principal component analysis [22], (ii) partial least squares regression [23], and (iii) linear discriminant analysis [24].

### 2.4. Learning Models

The presentation of the learning models in ML is limited to the ones that have been implemented in works presented in this review.

#### 2.4.1. Regression

Regression constitutes a supervised learning model, which aims to provide the prediction of an output variable according to the input variables, which are known. Most known algorithms include linear regression and logistic regression [25], as well as stepwise regression [26]. Also, more complex regression algorithms have been developed, such as ordinary least squares regression [27], multivariate adaptive regression splines [28], multiple linear regression, cubist [29], and locally estimated scatterplot smoothing [30].

#### 2.4.2. Clustering

Clustering [31] is a typical application of unsupervised learning model, typically used to find natural groupings of data (clusters). Well established clustering techniques are the k-means technique [32], the hierarchical technique [33], and the expectation maximisation technique [34].

#### 2.4.3. Bayesian Models

Bayesian models (BM) are a family of probabilistic graphical models in which the analysis is undertaken within the context of Bayesian inference. This type of model belongs to the supervised learning category and can be employed for solving either classification or regression problems. Naive bayes [35], gaussian naive bayes, multinomial naive bayes, bayesian network [36], mixture of gaussians [37], and bayesian belief network [38] are some of the most prominent algorithms in the literature.

#### 2.4.4. Instance Based Models

Instance based models (IBM) are memory-based models that learn by comparing new examples with instances in the training database. They construct hypotheses directly from the data available, while they do not maintain a set of abstractions, and generate classification or regression predictions using only specific instances. The disadvantage of these models is that their complexity grows with data. The most common learning algorithms in this category are the k-nearest neighbor [39], locally weighted learning [40], and learning vector quantization [41].

#### 2.4.5. Decision Trees

Decision trees (DT) are classification or regression models formulated in a tree-like architecture [42]. With DT, the dataset is progressively organized in smaller homogeneous subsets (sub-populations), while at the same time, an associated tree graph is generated. Each internal node of the tree structure represents a different pairwise comparison on a selected feature, whereas each branch represents the outcome of this comparison. Leaf nodes represent the final decision or prediction taken after following the path from root to leaf (expressed as a classification rule). The most common learning algorithms in this category are the classification and regression trees [43], the chi-square automatic interaction detector [44], and the iterative dichotomiser [45].

#### 2.4.6. Artificial Neural Networks

Artificial neural networks (ANNs) are divided into two categories; “Traditional ANNs” and “Deep ANNs”.

ANNs are inspired by the human brain functionality, emulating complex functions such as pattern generation, cognition, learning, and decision making [46]. The human brain consists of billions of neurons that inter-communicate and process any information provided. Similarly, an ANN as a simplified model of the structure of the biological neural network, consists of interconnected processing units organized in a specific topology. A number of nodes are arranged in multiple layers including the following:

1. An input layer where the data is fed into the system,
2. One or more hidden layers where the learning takes place, and
3. An output layer where the decision/prediction is given.

ANNs are supervised models that are typically used for regression and classification problems. The learning algorithms commonly used in ANNs include the radial basis function networks [47], perceptron algorithms [48], back-propagation [49], and resilient back-propagation [50]. Also, a large number of ANN-based learning algorithms have been reported, such as counter propagation algorithms [51], adaptive-neuro fuzzy inference systems [52], autoencoder, XY-Fusion, and supervised Kohonen networks [53], as well as Hopfield networks [54], multilayer perceptron [55], self-organising maps [56], extreme learning machines [57], generalized regression neural network [58], ensemble neural networks or ensemble averaging, and self-adaptive evolutionary extreme learning machines [59].

Deep ANNs are most widely referred to as deep learning (DL) or deep neural networks (DNNs) [60]. They are a relatively new area of ML research allowing computational models that are composed of multiple processing layers to learn complex data representations using multiple levels of abstraction. One of the main advantages of DL is that in some cases, the step of feature extraction is performed by the model itself. DL models have dramatically improved the state-of-the-art in many different sectors and industries, including agriculture. DNN’s are simply an ANN with multiple hidden layers between the input and output layers and can be either supervised, partially supervised, or even unsupervised. A common DL model is the convolutional neural network (CNN), where feature maps are extracted by performing convolutions in the image domain. A comprehensive introduction on CNNs is given in the literature [61]. Other typical DL architectures include deep Boltzmann machine, deep belief network [62], and auto-encoders [63].

#### 2.4.7. Support Vector Machines

Support vector machines (SVMs) were first introduced in the work of [64] on the foundation of statistical learning theory. SVM is intrinsically a binary classifier that constructs a linear separating hyperplane to classify data instances. The classification capabilities of traditional SVMs can be substantially enhanced through transformation of the original feature space into a feature space of a higher dimension by using the “kernel trick”. SVMs have been used for classification, regression, and clustering. Based on global optimization, SVMs deal with overfitting problems, which appear in high-dimensional spaces, making them appealing in various applications [65,66]. Most used SVM algorithms include the support vector regression [67], least squares support vector machine [68], and successive projection algorithm-support vector machine [69].

#### 2.4.8. Ensemble Learning

Ensemble learning (EL) models aim at improving the predictive performance of a given statistical learning or model fitting technique by constructing a linear combination of simpler base learner. Considering that each trained ensemble represents a single hypothesis, these multiple-classifier systems enable hybridization of hypotheses not induced by the same base learner, thus yielding better results in the case of significant diversity among the single models. Decision trees have been typically used as the base learner in EL models, for example, random forest [70], whereas a large number of boosting and bagging implementations have been also proposed, for example, boosting technique [71], adaboost [72], and bootstrap aggregating or bagging algorithm [73].

## 3. Review

The reviewed articles have been, on a first level, classified in four generic categories; namely, crop management, livestock management, water management, and soil management. The applications of ML in the crop section were divided into sub-categories including yield prediction, disease detection, weed detection crop quality, and species recognition. The applications of ML in the livestock section were divided into two sub-categories; animal welfare and livestock production.

The search engines implemented were Scopus, ScienceDirect and PubMed. The selected articles regard works presented solely in journal papers. Climate prediction, although very important for agricultural production, has not been included in the presented review, considering the fact that ML applications for climate prediction is a complete area by itself. Finally, all articles presented here regard the period from 2004 up to the present.

### 3.1. Crop Management

#### 3.1.1. Yield Prediction

Yield prediction, one of the most significant topics in precision agriculture, is of high importance for yield mapping, yield estimation, matching of crop supply with demand, and crop management to increase productivity. Examples of ML applications include in those in the works of [74]; an efficient, low-cost, and non-destructive method that automatically counted coffee fruits on a branch. The method calculates the coffee fruits in three categories: harvestable, not harvestable, and fruits with disregarded maturation stage. In addition, the method estimated the weight and the maturation percentage of the coffee fruits. The aim of this work was to provide information to coffee growers to optimise economic benefits and plan their agricultural work. Another study that used for yield prediction is that by the authors of [75], in which they developed a machine vision system for automating shaking and catching cherries during harvest. The system segments and detects occluded cherry branches with full foliage even when these are inconspicuous. The main aim of the system was to reduce labor requirements in manual harvesting and handling operations. In another study [76], authors developed an early yield mapping system for the identification of immature green citrus in a citrus grove under outdoor conditions. As all other relative studies, the aim of the study was to provide growers with yield-specific information to assist them to optimise their grove in terms of profit and increased yield. In another study [77], the authors developed a model for the estimation of grassland biomass (kg dry matter/ha/day) based on ANNs and multitemporal remote sensing data. Another study dedicated to yield prediction, and specifically to wheat yield prediction, was presented in another study [78]. The developed method used satellite imagery and received crop growth characteristics fused with soil data for a more accurate prediction. The authors of [79] presented a method for the detection of tomatoes based on EM and remotely sensed red green blue (RGB) images, which were captured by an unmanned aerial vehicle (UAV). Also, in the work of [80], authors developed a method for the rice development stage prediction based on SVM and basic geographic information obtained from weather stations in China. Finally, a generalized method for agricultural yield predictions, was presented in another study [81]. The method is based on an ENN application on long-period generated agronomical data (1997–2014). The study regards regional predictions (specifically in in Taiwan) focused on the supporting farmers to avoid imbalances in market supply and demand caused or hastened by harvest crop quality.

Table 5 summarizes the above papers for the case of yield prediction sub-category.

#### 3.1.2. Disease Detection

Disease detection and yield prediction are the sub-categories with the higher number of articles presented in this review. One of the most significant concerns in agriculture is pest and disease control in open-air (arable farming) and greenhouse conditions. The most widely used practice in pest and disease control is to uniformly spray pesticides over the cropping area. This practice, although effective, has a high financial and significant environmental cost. Environmental impacts can be residues in crop products, side effects on ground water contamination, impacts on local wildlife and eco-systems, and so on. ML is an integrated part of precision agriculture management, where agro-chemicals input is targeted in terms of time and place. In the literature [82], a tool is presented for the detection and discrimination of healthy *Silybum marianum* plants and those infected by smut fungus *Microbotyum silybum* during vegetative growth. In the work of [83], authors developed a new method based on image processing procedure for the classification of parasites and the automatic detection of thrips in strawberry greenhouse environment, for real-time control. The authos of [84] presented a method for detection and screening of Bakanae disease in rice seedlings. More specifically, the aim of the study was the accurate detection of pathogen *Fusarium fujikuroi* for two rice cultivars. The automated detection of infected plants increased grain yield and was less time-consuming compared with naked eye examination.

Wheat is one of the most economically significant crops worldwide. The last five studies presented in this sub-category are dedicated to the detection and discrimination between diseased and healthy wheat crops. The authors of [85] developed a new system for the detection of nitrogen stressed, and yellow rust infected and healthy winter wheat canopies based on hierarchical self-organizing classifier and hyperspectral reflectance imaging data. The study aimed at the accurate detection of these categories for a more effective usage of fungicides and fertilizers according to the plant’s needs. In the next case study [86], the development of a system was presented that automatically discriminated between water stressed *Septoria tritici* infected and healthy winter wheat canopies. The approach used an least squares (LS)-SVM classifier with optical multisensor fusion. The authors of [87] presented a method to detect either yellow rust infected or healthy wheat, based on ANN models and spectral reflectance features. The accurate detection of either infected or healthy plants enables the precise targeting of pesticides in the field. In the work of [88], a real time remote sensing system is presented for the detection of yellow rust infected and healthy wheat. The system is based on a self-organising map (SOM) neural network and data fusion of hyper-spectral reflection and multi-spectral fluorescence imaging. The goal of the study was the accurate detection, before it can visibly detected, of yellow rust infected winter wheat cultivar “Madrigal”. Finally, the authors of [89] presented a method for the simultaneous identification and discrimination of yellow rust infected, and nitrogen stressed and healthy wheat plants of cultivar “Madrigal”. The approach is based on an SOM neural network and hyperspectral reflectance imaging. The aim of the study was the accurate discrimination between the plant stress, which is caused by disease and nutrient deficiency stress under field conditions. Finally, the author of [90] presented a CNN-based method for the disease detection diagnosis based on simple leaves images with sufficient accuracy to classify between healthy and diseased leaves in various plants.

Table 6 summarizes the above papers for the case of the disease detection sub-category.

#### 3.1.3. Weed Detection

Weed detection and management is another significant problem in agriculture. Many producers indicate weeds as the most important threat to crop production. The accurate detection of weeds is of high importance to sustainable agriculture, because weeds are difficult to detect and discriminate from crops. Again, ML algorithms in conjunction with sensors can lead to accurate detection and discrimination of weeds with low cost and with no environmental issues and side effects. ML for weed detection can enable the development of tools and robots to destroy weeds, which minimise the need for herbicides. Two studies on ML applications for weed detection issues in agriculture have been presented. In the first study [91], authors presented a new method based on counter propagation (CP)-ANN and multispectral images captured by unmanned aircraft systems (UAS) for the identification of *Silybum marianum*, a weed that is hard to eradicate and causes major loss on crop yield. In the second study [92], the authors developed a new method based on ML techniques and hyperspectral imaging, for crop and weed species recognition. More specifically, the authors created an active learning system for the recognition of Maize (*Zea mayas*), as crop plant species and Ranunculus repens, Cirsium arvense, Sinapis arvensis, Stellaria media, Tarraxacum officinale, Poa annua, Polygonum persicaria, Urtica dioica, Oxalis europaea, and Medicago lupulina as weed species. The main goal was the accurate recognition and discrimination of these species for economic and environmental purposes. In another study [93], the authors developed a weed detection method based on SVN in grassland cropping.

Table 7 summarizes the above papers for the case of weed detection sub-category.

#### 3.1.4. Crop Quality

The penultimate sub-category for the crop category is studies developed for the identification of features connected with the crop quality. The accurate detection and classification of crop quality characteristics can increase product price and reduce waste. In the first study [94], the authors presented and developed a new method for the detection and classification of botanical and non-botanical foreign matter embedded inside cotton lint during harvesting. The aim of the study was quality improvement while the minimising fiber damage. Another study [95] regards pears production and, more specifically, a method was presented for the identification and differentiation of Korla fragrant pears into deciduous-calyx or persistent-calyx categories. The approach applied ML methods with hyperspectral reflectance imaging. The final study for this sub-category was by the authors of [96], in which a method was presented for the prediction and classification of the geographical origin for rice samples. The method was based on ML techniques applied on chemical components of samples. More specifically, the main goal was the classification of the geographical origin of rice, for two different climate regions in Brazil; Goias and Rio Grande do Sul. The results showed that Cd, Rb, Mg, and K are the four most relevant chemical components for the classification of samples.

Table 8 summarizes the above presented articles.

#### 3.1.5. Species Recognition

The last sub-category of crop category is the species recognition. The main goal is the automatic identification and classification of plant species in order to avoid the use of human experts, as well as to reduce the classification time. A method for the identification and classification of three legume species, namely, white beans, red beans, and soybean, via leaf vein patterns has been presented in [97]. Vein morphology carries accurate information about the properties of the leaf. It is an ideal tool for plant identification in comparison with color and shape.

Table 9 summarizes the above study for the case of species recognition sub-category.

### 3.2. Livestock Management

The livestock category consists of two sub-categories, namely, animal welfare and livestock production. Animal welfare deals with the health and wellbeing of animals, with the main application of ML in monitoring animal behaviour for the early detection of diseases. On the other hand, livestock production deals with issues in the production system, where the main scope of ML applications is the accurate estimation of economic balances for the producers based on production line monitoring.

#### 3.2.1. Animal Welfare

Several articles are reported to belong to the animal welfare sub-category. In the first article [98], a method is presented for the classification of cattle behaviour based on ML models using data collected by collar sensors with magnetometers and three-axis accelerometers. The aim of the study was the prediction of events such as the oestrus and the recognition of dietary changes on cattle. In the second article [99], a system was presented for the automatic identification and classification of chewing patterns in calves. The authors created a system based on ML applying data from chewing signals of dietary supplements, such as hay and ryegrass, combined with behaviour data, such as rumination and idleness. Data was collected by optical FBG sensors. In another study [100], an automated monitoring system based on ML was presented for animal behavior tracking, including tracking of animal movements by depth video cameras, for monitoring various activities of the animal (standing, moving, feeding, and drinking).

Table 10 summarizes the features of the above presented articles.

#### 3.2.2. Livestock Production

The sub-category of livestock production regards studies developed for the accurate prediction and estimation of farming parameters to optimize the economic efficiency of the production system. This sub-category consists of the presentation of four articles, three with cattle production and one for hens’ eggs production. In the work of [101], a method for the prediction of the rumen fermentation pattern from milk fatty acids was presented. The main aim of the study was to achieve the most accurate prediction of rumen fermentations, which play a significant role for the evaluation of diets for milk production. In addition, this work showed that milk fatty acids have ideal features to predict the molar proportions of volatile fatty acids in the rumen. The next study [102] was related to hen production. Specifically, a method based on SVM model was presented for the early detection and warning of problems in the commercial production of eggs. Based on SVM methods [103], a method for the accurate estimation of bovine weight trajectories over time was presented. The accurate estimation of cattle weights is very important for breeders. The last article of the section [104] deals with the development of a function for the prediction of carcass weight for beef cattle of the Asturiana de los Valles breed based on SVR models and zoometric measurements features. The results show that the presented method can predict carcass weights 150 days prior to the slaughter day. The authors of [105] presented a method based on convolutional neural networks (CNNs) applied in digital images for pig face recognition. The main aim of the research was the identification of animals without the need for radio frequency identification (RFID) tags, which involve a distressing activity for the animal, are limited in their range, and are a time-consuming method.

Table 11 summarizes the features of the above presented works.

### 3.3. Water Management

Water management in agricultural production requires significant efforts and plays a significant role in hydrological, climatological, and agronomical balance.

This section consists of four studies that were mostly developed for the estimation of daily, weekly, or monthly evapotranspiration. The accurate estimation of evapotranspiration is a complex process and is of a high importance for resource management in crop production, as well as for the design and the operation management of irrigation systems. In another study [106], the authors developed a computational method for the estimation of monthly mean evapotranspiration for arid and semi-arid regions. It used monthly mean climatic data of 44 meteorological stations for the period 1951–2010. In another study dedicated to ML applications on agricultural water management [107], two scenarios were presented for the estimation of the daily evapotranspiration from temperature data collected from six meteorological stations of a region during the long period (i.e., 1961–2014). Finally, in another study [108], authors developed a method based on ELM model fed with temperature data for the weekly estimation of evapotranspiration for two meteorological weather stations. The purpose was the accurate estimation of weekly evapotranspiration in arid regions of India based on limited data scenario for crop water management.

Daily dew point temperature, on the other hand, is a significant element for the identification of expected weather phenomena, as well as for the estimation of evapotranspiration and evaporation. In another article [109], a model is presented for the prediction of daily dew point temperature, based on ML. The weather data were collected from two different weather stations.

Table 12 summarizes the above papers for the case of the water management sub-category.

### 3.4. Soil Management

The final category of this review concerns ML application on prediction-identification of agricultural soil properties, such as the estimation of soil drying, condition, temperature, and moisture content. Soil is a heterogeneous natural resource, with complex processes and mechanisms that are difficult to understand. Soil properties allow researchers to understand the dynamics of ecosystems and the impingement in agriculture. The accurate estimation of soil conditions can lead to improved soil management. Soil temperature alone plays a significant role for the accurate analysis of the climate change effects of a region and eco-environmental conditions. It is a significant meteorological parameter controlling the interactive processes between ground and atmosphere. In addition, soil moisture has an important role for crop yield variability. However, soil measurements are generally time-consuming and expensive, so a low cost and reliable solution for the accurate estimation of soil can be achieved with the usage of computational analysis based on ML techniques. The first study for this last sub-category is the work of [110]. More specifically, this study presented a method for the evaluation of soil drying for agricultural planning. The method accurately evaluates the soil drying, with evapotranspiration and precipitation data, in a region located in Urbana, IL of the United States. The goal of this method was the provision of remote agricultural management decisions. The second study [111] was developed for the prediction of soil condition. In particular, the study presented the comparison of four regression models for the prediction of soil organic carbon (OC), moisture content (MC), and total nitrogen (TN). More specifically, the authors used a visible-near infrared (VIS-NIR) spectrophotometer to collect soil spectra from 140 unprocessed and wet samples of the top layer of Luvisol soil types. The samples were collected from an arable field in Premslin, Germany in August 2013, after the harvest of wheat crops. They concluded that the accurate prediction of soil properties can optimize soil management. In a third study [112], the authors developed a new method based on a self adaptive evolutionary-extreme learning machine (SaE-ELM) model and daily weather data for the estimation of daily soil temperature at six different depths of 5, 10, 20, 30, 50, and 100 cm in two different in climate conditions regions of Iran; Bandar Abbas and Kerman. The aim was the accurate estimation of soil temperature for agricultural management. The last study [113] presented a novel method for the estimation of soil moisture, based on ANN models using data from force sensors on a no-till chisel opener.

Table 13 summarizes the above papers for the case of soil management sub-category.

## 4. Discussion and Conclusions

**Figure 2:**
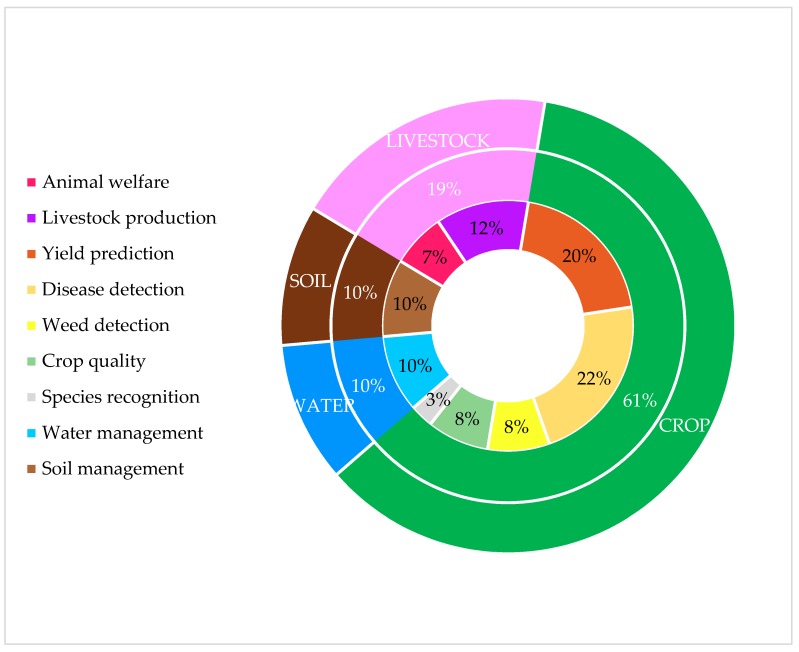
Pie chart presenting the papers according to the application domains.

The number of articles included in this review was 40 in total. Twenty-five (25) of the presented articles were published in the journal «Computer and Electronics in Agriculture», six were published in the journal of «Biosystems Engineering», and the rest of the articles were published to the following journals: «Sensors», «Sustainability», «Real-Time Imagining», «Precision Agriculture», «Earth Observations and Remote Sensing», «Saudi Journal of Biological Sciences», «Scientific Reports», and «Computers in Industry». Among the articles, eight of them are related to applications of ML in livestock management, four articles are related to applications of ML in water management, four are related to soil management, while the largest number of them (i.e., 24 articles) are related to applications of ML in crop management. Figure 2 presents the distribution of the articles according to these application domains and to the defined sub-categories.

**Figure 3:**
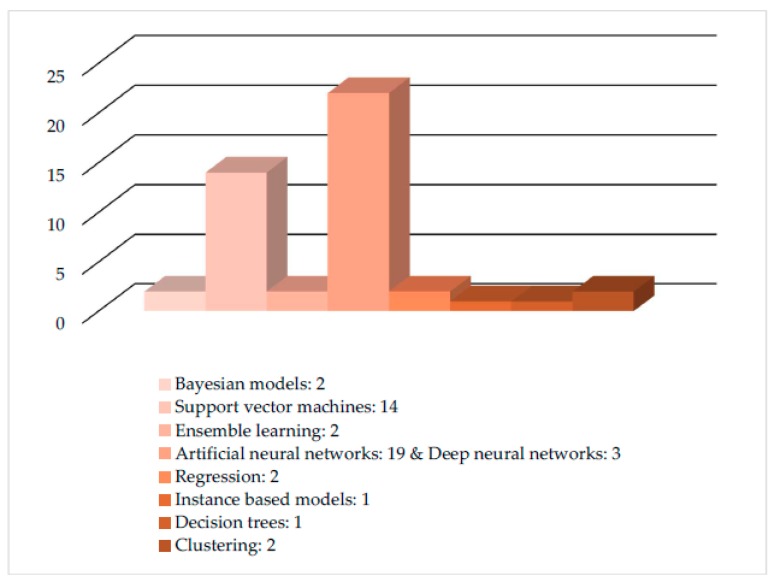
Presentation of machine learning (ML) models with their total rate.

**Figure 4:**
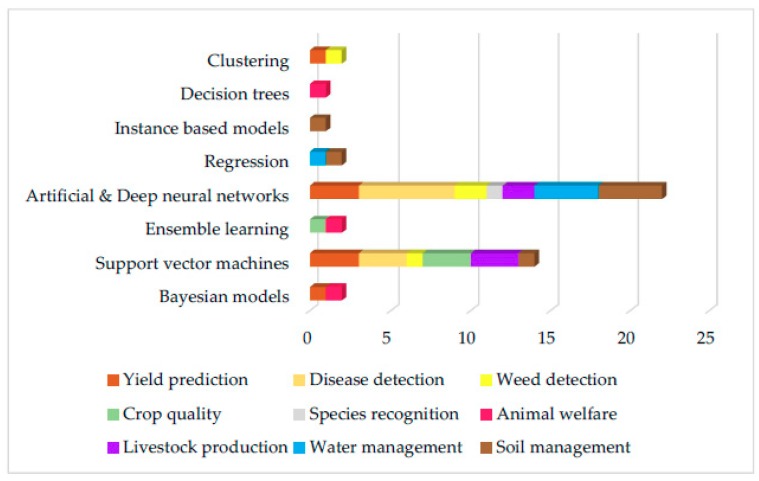
The total number of ML models according to each sub-category of the four main categories.

**Figure 5:**
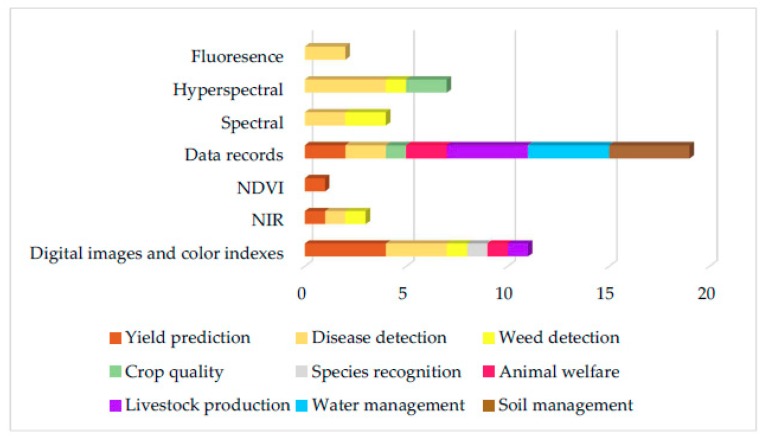
Data resources usage according to each sub-category. NDVI—normalized difference vegetation index; NIR—near infrared.

From the analysis of these articles, it was found that eight ML models have been implemented in total. More specifically, five ML models were implemented in the approaches on crop management, where the most popular models were ANNs (with most frequent crop at hand—wheat). In livestock management category, four ML models were implemented, with most popular models being SVMs (most frequent livestock type at hand—cattle). For water management in particular evapotranspiration estimation, two ML models were implemented and the most frequently implemented were ANNs. Finally, in the soil management category, four ML models were implemented, with the most popular one again being the ANN model. In Figure 3, the eight ML models with their total rates are presented, and in Figure 4 and Table 14, the ML models for all studies according to the sub-category are presented. Finally, in Figure 5 and Table 15, the future techniques that were used according to each sub-category are presented (it is noting that the figure and table provide the same information in different demonstration purposes).

From the above figures and tables, we show that ML models have been applied in multiple applications for crop management (61%); mostly yield prediction (20%) and disease detection (22%). This trend in the applications distribution reflects the data intense applications within crop and high use of images (spectral, hyperspectral, NIR, etc.). Data analysis, as a mature scientific field, provides the ground for the development of numerous applications related to crop management because, in most cases, ML-based predictions can be extracted without the need for fusion of data from other resources. In contrast, when data recordings are involved, occasionally at the level of big data, the implementations of ML are less in number, mainly because of the increased efforts required for the data analysis task and not for the ML models per se. This fact partially explains the almost equal distribution of ML applications in livestock management (19%), water management (10%), and soil management (10%). It is also evident from the analysis that most of the studies used ANN and SVM ML models. More specifically, ANNs were used mostly for implementations in crop, water, and soil management, while SVMs were used mostly for livestock management.

By applying machine learning to sensor data, farm management systems are evolving into real artificial intelligence systems, providing richer recommendations and insights for the subsequent decisions and actions with the ultimate scope of production improvement. For this scope, in the future, it is expected that the usage of ML models will be even more widespread, allowing for the possibility of integrated and applicable tools. At the moment, all of the approaches regard individual approaches and solutions and are not adequately connected with the decision-making process, as seen in other application domains. This integration of automated data recording, data analysis, ML implementation, and decision-making or support will provide practical tolls that come in line with the so-called knowledge-based agriculture for increasing production levels and bio-products quality.

## Figures and Tables

**Table 1:** Abbreviations for machine learning models.

Abbreviation	Model
ANNs	artificial neural networks
BM	bayesian models
DL	deep learning
DR	dimensionality reduction
DT	decision trees
EL	ensemble learning
IBM	instance based models
SVMs	support vector machines

**Table 2:** Abbreviations for machine learning algorithms.

Abbreviation	Algorithm
ANFIS	adaptive-neuro fuzzy inference systems
Bagging	bootstrap aggregating
BBN	bayesian belief network
BN	bayesian network
BPN	back-propagation network
CART	classification and regression trees
CHAID	chi-square automatic interaction detector
CNNs	convolutional neural networks
CP	counter propagation
DBM	deep boltzmann machine
DBN	deep belief network
DNN	deep neural networks
ELMs	extreme learning machines
EM	expectation maximisation
ENNs	ensemble neural networks
GNB	gaussian naive bayes
GRNN	generalized regression neural network
KNN	k-nearest neighbor
LDA	linear discriminant analysis
LS-SVM	least squares-support vector machine
LVQ	learning vector quantization
LWL	locally weighted learning
MARS	multivariate adaptive regression splines
MLP	multi-layer perceptron
MLR	multiple linear regression
MOG	mixture of gaussians
OLSR	ordinary least squares regression
PCA	principal component analysis
PLSR	partial least squares regression
RBFN	radial basis function networks
RF	random forest
SaE-ELM	self adaptive evolutionary-extreme learning machine
SKNs	supervised kohonen networks
SOMs	self-organising maps
SPA-SVM	successive projection algorithm-support vector machine
SVR	support vector regression

**Table 3:** Abbreviations for statistical measures for the validation of machine learning algorithms.

Abbreviation	Measure
APE	average prediction error
MABE	mean absolute bias error
MAE	mean absolute error
MAPE	mean absolute percentage error
MPE	mean percentage error
NS	nash-sutcliffe coefficient
R	radius
R^2^	coefficient of determination
RMSE	root mean squared error
RMSEP	root mean square error of prediction
RPD	relative percentage difference
RRMSE	average relative root mean square error

**Table 4:** General abbreviations.

Abbreviation	
AUS	aircraft unmanned system
Cd	cadmium
FBG	fiber bragg grating
HSV	hue saturation value color space
K	potassium
MC	moisture content
Mg	magnesium
ML	machine learning
NDVI	normalized difference vegetation index
NIR	near infrared
OC	organic carbon
Rb	rubidium
RGB	red green blue
TN	total nitrogen
UAV	unmanned aerial vehicle
VIS-NIR	visible-near infrared

**Table 5:** Crop: yield prediction table.

Article	Crop	Observed Features	Functionality	Models/Algorithms	Results
[74]	Coffee	Forty-two (42) color features in digital images illustrating coffee fruits	Automatic count of coffee fruits on a coffee branch	SVM	Harvestable: (1) Ripe/overripe: 82.54–87.83% visibility percentage (2) Semi-ripe: 68.25–85.36% visibility percentage Not harvestable: (1) Unripe: 76.91–81.39% visibility percentage
[75]	Cherry	Colored digital images depicting leaves, branches, cherry fruits, and the background	Detection of cherry branches with full foliage	BM/GNB	89.6% accuracy
[76]	Green citrus	Image features (form 20 × 20 pixels digital images of unripe green citrus fruits) such as coarseness, contrast, directionality, line-likeness, regularity, roughness, granularity, irregularity, brightness, smoothness, and fineness	Identification of the number of immature green citrus fruit under natural outdoor conditions	SVM	80.4% accuracy
[77]	Grass	Vegetation indices, spectral bands of red and NIR	Estimation of grassland biomass (kg dry matter/ha/day) for two managed grassland farms in Ireland; Moorepark and Grange	ANN/ANFIS	Moorepark: R^2^ = 0.85 RMSE = 11.07 Grange: R^2^ = 0.76 RMSE = 15.35
[78]	Wheat	Normalized values of on-line predicted soil parameters and the satellite NDVI	Wheat yield prediction within field variation	ANN/SNKs	81.65% accuracy
[79]	Tomato	High spatial resolution RGB images	Detection of tomatoes via RGB images captured by UAV	Clustering/EM	Recall: 0.6066 Precision: 0.9191 F-Measure: 0.7308
[80]	Rice	Agricultural, surface weather, and soil physico-chemical data with yield or development records	Rice development stage prediction and yield prediction	SVM	Middle-season rice: Tillering stage: RMSE (kg h^−1^ m^2^) = 126.8 Heading stage: RMSE (kg h^−1^ m^2^) = 96.4 Milk stage: RMSE (kg h^−1^ m^2^) = 109.4 Early rice: Tillering stage: RMSE (kg h^−1^ m^2^) = 88.3 Heading stage: RMSE (kg h^−1^ m^2^) = 68.0 Milk stage: RMSE (kg h^−1^ m^2^) = 36.4 Late rice: Tillering stage: RMSE (kg h^−1^ m^2^) = 89.2 Heading stage: RMSE (kg h^−1^ m^2^) = 69.7 Milk stage: RMSE (kg h^−1^ m^2^) = 46.5
[81]	General	Agriculture data: meteorological, environmental, economic, and harvest	Method for the accurate analysis for agricultural yield predictions	ANN/ENN and BPN based	1.3% error rate

**Table 6:** Crop: disease detection table.

Author	Crop	Observed Features	Functionality	Models/Algorithms	Results
[82]	*Silybum marianum*	Images with leaf spectra using a handheld visible and NIR spectrometer	Detection and discrimination between healthy *Silybum marianum* plants and those that are infected by smut fungus *Microbotyum silybum*	ANN/XY-Fusion	95.16% accuracy
[83]	Strawberry	Region index: ratio of major diameter to minor diameter; and color indexes: hue, saturation, and intensify	Classification of parasites and automatic detection of thrips	SVM	MPE = 2.25%
[84]	Rice	Morphological and color traits from healthy and infected from Bakanae disease, rice seedlings, for cultivars Tainan 11 and Toyonishiki	Detection of Bakanae disease, *Fusarium fujikuroi*, in rice seedlings	SVM	87.9% accuracy
[85]	Wheat	Hyperspectral reflectance imaging data	Detection of nitrogen stressed, yellow rust infected and healthy winter wheat canopies	ANN/XY-Fusion	Nitrogen stressed: 99.63% accuracy Yellow rust: 99.83% accuracy Healthy: 97.27% accuracy
[86]	Wheat	Spectral reflectance and fluorescence features	Detection of water stressed, *Septoria tritici* infected, and healthy winter wheat canopies	SVM/LS-SVM	Four scenarios: (1) Control treatment, healthy and well supplied with water: 100% accuracy (2) Inoculated treatment, with *Septoria tritici* and well supplied with water: 98.75% accuracy (3) Healthy treatment and deficient water supply: 100% accuracy (4) Inoculated treatment and deficient water supply: 98.7% accuracy
[87]	Wheat	Spectral reflectance features	Detection of yellow rust infected and healthy winter wheat canopies	ANN/MLP	Yellow rust infected wheat: 99.4% accuracy Healthy: 98.9% accuracy
[88]	Wheat	Data fusion of hyper-spectral reflection and multi-spectral fluorescence imaging	Detection of yellow rust infected and healthy winter wheat under field circumstances	ANN/SOM	Yellow rust infected wheat: 99.4% accuracy Healthy: 98.7% accuracy
[89]	Wheat	Hyperspectral reflectance images	Identification and discrimination of yellow rust infected, nitrogen stressed, and healthy winter wheat in field conditions	ANN/SOM	Yellow rust infected wheat: 99.92% accuracy Nitrogen stressed: 100% accuracy Healthy: 99.39% accuracy
[90]	Generilized approach for various crops (25 in total)	Simple leaves images of healthy and diseased plants	Detection and diagnosis of plant diseases	DNN/CNN	99.53% accuracy

**Table 7:** Crop: Weed detection table.

Author	Observed Features	Functionality	Models/Algorithms	Results
[91]	Spectral bands of red, green, and NIR and texture layer	Detection and mapping of *Silybum marianum*	ANN/CP	98.87% accuracy
[92]	Spectral features from hyperspectral imaging	Recognition and discrimination of *Zea mays* and weed species	ANN/one-class SOM and Clustering/one-class MOG	*Zea mays*: SOM = 100% accuracy MOG = 100% accuracy Weed species: SOM = 53–94% accuracy MOG = 31–98% accuracy
[93]	Camera images of grass and various weeds types	Reporting on performance of classification methods for grass vs. weed detection	SVN	97.9% Again Rumex classification6 94.65% Urtica classification 95.1% for mixed weed and mixed weather conditions

**Table 8:** Crop: crop quality table.

Author	Crop	Observed Features	Functionality	Models/Algorithms	Results
[94]	Cotton	Short wave infrared hyperspectral transmittance images depicting cotton along with botanical and non-botanical types of foreign matter	Detection and classification of common types of botanical and non-botanical foreign matter that are embedded inside the cotton lint	SVM	According to the optimal selected wavelengths, the classification accuracies are over 95% for the spectra and the images.
[95]	Pears	Hyperspectral reflectance imaging	Identification and differentiation of Korla fragrant pears into deciduous-calyx or persistent-calyx categories	SVM/SPA-SVM	Deciduous-calyx pears: 93.3% accuracy Persistent-calyx pears: 96.7% accuracy
[96]	Rice	Twenty (20) chemical components that were found in composition of rice samples with inductively coupled plasma mass spectrometry	Prediction and classification of geographical origin of a rice sample	EL/RF	93.83% accuracy

**Table 9:** Crop: Species recognition.

Author	Crop	Observed Features	Functionality	Models/Algorithms	Results
[97]	Legume	Vein leaf images of white and red beans as well as and soybean	Identification and classification of three legume species: soybean, and white and red bean	DL/CNN	White bean: 90.2% accuracy Red bean: 98.3% accuracy Soybean: 98.8% accuracy for five CNN layers

**Table 10:** Livestock: animal welfare.

Author	Animal Species	Observed Features	Functionality	Models/Algorithms	Results
[98]	Cattle	Features like grazing, ruminating, resting, and walking, which were recorded using collar systems with three-axis accelerometer and magnetometer	Classification of cattle behaviour	EL/Bagging with tree learner	96% accuracy
[99]	Calf	Data: chewing signals from dietary supplement, Tifton hay, ryegrass, rumination, and idleness. Signals were collected from optical FBG sensors	Identification and classification of chewing patterns in calves	DT/C4.5	94% accuracy
[100]	Pigs	3D motion data by using two depth cameras	Animal tracking and behavior annotation of the pigs to measure behavioral changes in pigs for welfare and health monitoring	BM: Gaussian Mixture Models (GMMs)	Animal tracking: mean multi-object tracking precision (MOTP) = 0.89 accuracy behavior annotation: standing: control R^2^ = 0.94, treatment R^2^ = 0.97 feeding: control R^2^ = 0.86, treatment R^2^ = 0.49

**Table 11:** Livestock: livestock production table.

Author	Animal Species	Observed Features	Functionality	Models/Algorithms	Results
[101]	Cattle	Milk fatty acids	Prediction of rumen fermentation pattern from milk fatty acids	ANN/BPN	Acetate: RMSE = 2.65% Propionate: RMSE = 7.67% Butyrate: RMSE = 7.61%
[102]	Hens	Six (6) features, which were created from mathematical models related to farm’s egg production line and collected over a period of seven (7) years.	Early detection and warning of problems in production curves of commercial hens eggs	SVM	98% accuracy
[103]	Bovine	Geometrical relationships of the trajectories of weights along the time	Estimation of cattle weight trajectories for future evolution with only one or a few weights.	SVM	Angus bulls from Indiana Beef Evaluation Program: weights 1, MAPE = 3.9 + −3.0% Bulls from Association of Breeder of Asturiana de los Valles: weights 1, MAPE = 5.3 + −4.4% Cow from Wokalup Selection Experiment in Western Australia: weights 1, MAPE = 9.3 + −6.7%
[104]	Cattle	Zoometric measurements of the animals 2 to 222 days before the slaughter	Prediction of carcass weight for beef cattle 150 days before the slaughter day	SVM/SVR	Average MAPE = 4.27%
[105]	Pigs	1553 color images with pigs faces	Pigs face recognition	DNNs: Convolutional Neural Networks (CNNs)	96.7% Accuracy

**Table 12:** Water: Water management table.

Author	Property	Observed Features	Functionality	Models/Algorithms	Results
[106]	Evapotranspiration	Data such as maximum, minimum, and mean temperature; relative humidity; solar radiation; and wind speed	Estimation of monthly mean reference evapotranspiration arid and semi-arid regions	Regression/MARS	MAE = 0.05 RMSE = 0.07 R = 0.9999
[107]	Evapotranspiration	Temperature data: maximum and minimum temperature, air temperature at 2 m height, mean relative humidity, wind speed at 10 m height, and sunshine duration	Estimation of daily evapotranspiration for two scenarios (six regional meteorological stations). Scenario A: Models trained and tested from local data of each Station (2). Scenario B: Models trained from pooled data from all stations	(1) Scenario ANN/ELM (2) Scenario ANN/GRNN	(1) Scenario A: RRMSE = 0.198 MAE = 0.267 mm d^−1^ NS = 0.891 (2) Scenario B: RRMSE = 0.194 MAE = 0.263 mm d^−1^ NS = 0.895
[108]	Evapotranspiration	Locally maximum and minimum air temperature, extraterrestrial radiation, and extrinsic evapotranspiration	Estimation of weekly evapotranspiration based on data from two meteorological weather stations	ANN/ELM	Station A: RMSE = 0.43 mm d^−1^ Station B: RMSE = 0.33 mm d^−1^
[109]	Daily dew point temperature	Weather data such as average air temperature, relative humidity, atmospheric pressure, vapor pressure, and horizontal global solar radiation	Prediction of daily dew point temperature	ANN/ELM	Region case A: MABE = 0.3240 °C RMSE = 0.5662 °C R = 0.9933 Region case B: MABE = 0.5203 °C RMSE = 0.6709 °C R = 0.9877

**Table 13:** Soil management table.

Author	Property	Observed Features	Functionality	Models/Algorithms	Results
[110]	Soil drying	Precipitation and potential evapotranspiration data	Evaluation of soil drying for agricultural planning	IBM/KNN and ANN/BP	Both performed with 91–94% accuracy
[111]	Soil condition	140 soil samples from top soil layer of an arable field	Prediction of soil OC, MC, and TN	SVM/LS-SVM and Regression/Cubist	OC: RMSEP = 0.062% & RPD = 2.20 (LS-SVM) MC: RMSEP = 0.457% & RPD = 2.24 (LS-SVM) TN: RMSEP = 0.071% & RPD = 1.96 (Cubist)
[112]	Soil temperature	Daily weather data: maximum, minimum, and average air temperature; global solar radiation; and atmospheric pressure. Data were collected for the period of 1996–2005 for Bandar Abbas and for the period of 1998–2004 for Kerman	Estimation of soil temperature for six (6) different depths 5, 10, 20, 30, 50, and 100 cm, in two different in climate conditions Iranian regions; Bandar Abbas and Kerman	ANN/SaE-ELM	Bandar Abbas station: MABE = 0.8046 to 1.5338 °C RMSE = 1.0958 to 1.9029 °C R = 0.9084 to 0.9893 Kerman station: MABE = 1.5415 to 2.3422 °C RMSE = 2.0017 to 2.9018 °C R = 0.8736 to 0.9831 depending on the depth
[113]	Soil moisture	Dataset of forces acting on a chisel and speed	Estimation of soil moisture	ANN/MLP and RBF	MLP: RMSE = 1.27% R^2^ = 0.79 APE = 3.77% RBF: RMSE = 1.30% R^2^ = 0.80 APE = 3.75%

**Table 14:** The total number of ML models according to each sub-category of the four main categories.

ML Models Per Section									
Model	Crop					Livestock		Water	Soil
	Yield Prediction	Disease Detection	Weed Detection	Crop Quality	Species Recognition	Animal Welfare	Livestock Production	Water Management	Soil Management
Bayesian models	**1**					**1**			
Support vector machines	**3**	**3**	**1**	**3**			**3**		**1**
Ensemble learning				**1**		**1**			
Artificial & Deep neural networks	**3**	**6**	**2**		**1**		**2**	**4**	**4**
Regression								**1**	**1**
Instance based models									**1**
Decision trees						**1**			
Clustering	**1**		**1**						
Total	**8**	**9**	**4**	**4**	**1**	**3**	**5**	**5**	**7**

**Table 15:** Data resources usage according to each sub-category.

Feature Collection									
**Feature Technique**	Crop					Livestock		Water	Soil
	Yield Prediction	Disease Detection	Weed Detection	Crop Quality	Species recognition	Animal Welfare	Livestock Production	Water Management	Soil Management
Digital images and color indexes	4	3	1		1	1	1		
NIR	1	1	1						
NDVI	1								
Data records	2	2		1		2	4	4	4
Spectral		2	2						
Hyperspectral		4	1	2					
Fluoresence		2							
